# Membrane-type I matrix metalloproteinase (MT1-MMP), lipid metabolism, and therapeutic implications

**DOI:** 10.1093/jmcb/mjab048

**Published:** 2021-07-23

**Authors:** Xiao-Dan Xia, Adekunle Alabi, Maggie Wang, Hong-Mei Gu, Rui Zhe Yang, Gui-Qing Wang, Da-Wei Zhang

**Affiliations:** 1 Department of Orthopedics, The Sixth Affiliated Hospital (Qingyuan People’s Hospital), Guangzhou Medical University, Qingyuan 511500, China; 2 Department of Pediatrics and Group on the Molecular and Cell Biology of Lipids, Faculty of Medicine and Dentistry, University of Alberta, Edmonton, AB T6R 2G3, Canada

**Keywords:** matrix metalloproteinase, low-density lipoprotein receptor, extracellular matrix, atherosclerosis, cardiovascular disease, obesity

## Abstract

Lipids exert many essential physiological functions, such as serving as a structural component of biological membranes, storing energy, and regulating cell signal transduction. Dysregulation of lipid metabolism can lead to dyslipidemia related to various human diseases, such as obesity, diabetes, and cardiovascular disease. Therefore, lipid metabolism is strictly regulated through multiple mechanisms at different levels, including the extracellular matrix. Membrane-type I matrix metalloproteinase (MT1-MMP), a zinc-dependent endopeptidase, proteolytically cleaves extracellular matrix components, and non-matrix proteins, thereby regulating many physiological and pathophysiological processes. Emerging evidence supports the vital role of MT1-MMP in lipid metabolism. For example, MT1-MMP mediates ectodomain shedding of low-density lipoprotein receptor and increases plasma low-density lipoprotein cholesterol levels and the development of atherosclerosis. It also increases the vulnerability of atherosclerotic plaque by promoting collagen cleavage. Furthermore, it can cleave the extracellular matrix of adipocytes, affecting adipogenesis and the development of obesity. Therefore, the activity of MT1-MMP is strictly regulated by multiple mechanisms, such as autocatalytic cleavage, endocytosis and exocytosis, and post-translational modifications. Here, we summarize the latest advances in MT1-MMP, mainly focusing on its role in lipid metabolism, the molecular mechanisms regulating the function and expression of MT1-MMP, and their pharmacotherapeutic implications.

## Introduction

Dyslipidemia is a critical factor in the development of various human diseases, such as cardiovascular disease, diabetes, and non-alcoholic fatty liver disease. Therefore, lipid homeostasis in humans is strictly regulated by a well-balanced mechanism of intestinal uptake, endogenous synthesis and metabolism, and transport in lipoprotein particles and excretion. It has recently been shown that membrane-type I matrix metalloproteinase (MT1-MMP/MMP14) plays an important role in regulating lipid metabolism by cleaving the extracellular matrix (ECM) and non-matrix proteins. Therefore, it becomes an excellent target for developing new therapies to treat dyslipidemia in humans.

**Figure 1:**
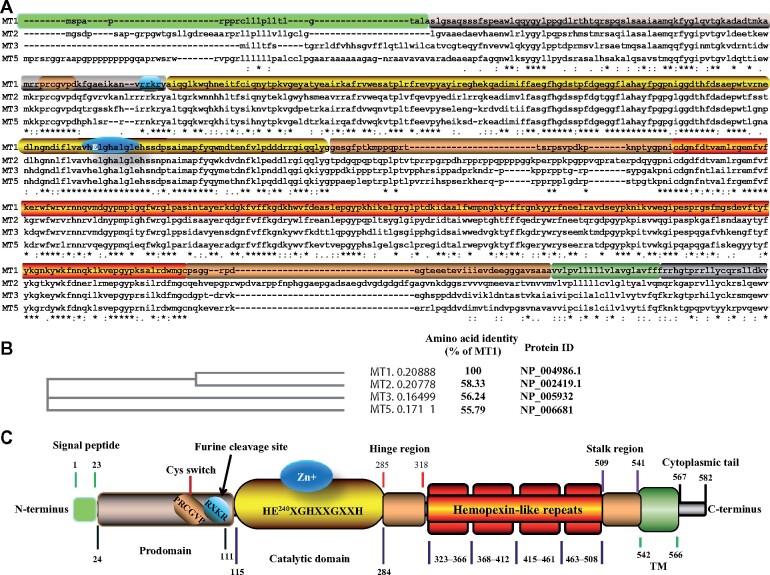
Structure of MT1-MMP. (**A**) Sequence alignment in CLUSTAL format. The alignment was performed using Multiple Alignment using Fast Fourier Transform (MAFFT)FFT-NS-I (v7.429). MT1-MMP: NP_004986; MT2-MMP: NP_002419.1; MT3-MMP: NP_005932; MT5-MMP: NP_006681. (**B**) Phylogenetic Tree of MT1-MMP, MT2-MMP, MT3-MMP, and MT5-MMP. Branch length is shown in the cladogram. Numbers next to each MT-MMP indicate the actual branch length. (**C**) A schematic of MT1-MMP. Protein functional motifs are indicated in different colors. TM, transmembrane domain.

MT1-MMP was discovered by Sato et al. (1994) in 1994. It belongs to the membrane-type subclass of the MMP family that includes four type I transmembrane MMPs (MT1-MMP/MMP14, MT2-MMP/MMP15, MT3-MMP/MMP16, and MT5-MMP/MMP24) and two glycosyl phosphatidylinositol-anchored membrane-associated MMPs (MT4-MMP/MMP17 and MT6-MMP/MMP25) (Itoh, 2015; Amar et al., 2017). Among the four transmembrane MMPs, MT2-MMP is the closest member to MT1-MMP, with amino acid identity of ∼58% (Figure 1A and B). This review focuses on the molecular mechanisms of the function and regulation of MT1-MMP, with a specific emphasis on recent research progress in the role of MT1-MMP in lipid metabolism, which may pave the way toward the development of potential novel clinical interventions for dyslipidemia and related human diseases.

## MT1-MMP, lipid metabolism, and relevant human diseases

### Structure and function of MT1-MMP

Human and mouse MT1-MMP proteins are encoded by the *MMP14/Mmp14* gene located at chromosome 14q11.2 and 14C2, respectively, containing 10 exons that encode a 582-amino acid MT1-MMP protein (Sato et al., 1994; Houghton, 2015). As shown in Figure 1C, MT1-MMP is composed of a series of functional domains, including an N-terminal signal peptide, a pro-peptide domain containing a conserved cysteine switch motif PRC^93^GVPD and a conserved R^108^XKR motif for recognition and cleavage by furin-like enzymes in the Golgi apparatus, and a catalytic domain containing the highest conserved core enzyme with a conserved zinc-binding site (HE^240^XGHXXGXXH). MT1-MMP also contains a flexible hinge region that links the catalytic domain and C-terminal domains, i.e. a hemopexin domain, a second linker or the stalk region, a transmembrane domain that anchors the protein to cell membranes, and a cytoplasmic tail (Houghton, 2015).

MT1-MMP plays an essential role in numerous fundamental physiological and pathophysiological processes, such as promoting angiogenesis, cell migration, tumor growth, and metastasis, modulating inflammation response and osteoclast activity, regulating the development of the early brain and neuromuscular junctions, and mediating the formation of intrahepatic bile ducts (Gifford and Itoh, 2019; Chan et al., 2020; Esteban et al., 2020; Jiang et al., 2020; Oentaryo et al., 2020; Sato et al., 2020; Zhu et al., 2020). Among all the MMP knockout mice that have been developed, only MT1-MMP global knockout mice (*Mmp14^−/−^*) display many serious adverse effects, such as skeletal dysplasia, craniofacial dysmorphism, severe osteopenia, and dwarfism. *Mmp14^−/−^* mice die at 3‒4 weeks after birth, indicating its indispensable role in postnatal development (Holmbeck et al., 1999; Zhou et al., 2000). Mutations in MT1-MMP are associated with Winchester syndrome in humans (de Vos et al., 2018). A variant in MT1-MMP (rs1042704) significantly reduces its collagenolytic activity, and it is causatively associated with Dupuytren’s disease (Itoh et al., 2021), while variants, rs1003349 and rs1004030, in the promoter region of the *MMP14* gene significantly increase its expression and are associated with an increased risk of gallbladder cancer in Indians (Vinay et al., 2021).

Unlike secreted MMPs, MT1-MMP localizes in specific domains within the plasma membrane, making it a significant advantage over soluble MMPs in proteolytical cleavage of peripheral substrates. MT1-MMP plays a pivotal role in tissue remodeling by directly degrading ECM components such as collagen, gelatin, vitronectin, laminin 1, and fibronectin, specifically activating proMMP2 and proMMP13, and cleaving a broad spectrum of non-matrix substrates (Sato et al., 1994; Knauper et al., 1996; Gifford and Itoh, 2019). In addition, a cell-based proteomic study using the isotope-coded affinity tagging technique has shown that MT1-MMP proteolytically cleaved >50 intracellular proteins involved in various physiological processes. A few examples include Niemann‒Pick disease type C2 protein, heat shock protein 90α, progranulin, galectin-1, and enolase-γ; some of them are further confirmed by an *in vitro* cleavage assay (Cauwe and Opdenakker, 2010). The levels of fructose-bisphosphate aldolase A, a cytoplasmic protein that plays an important role in glucose metabolism, were increased in mammary gland tissues of 1- to 2-week-old *Mmp14^−/−^* mice compared to that of the wild-type littermates (Mori et al., 2016). Overall, these findings indicate that, in addition to the extracellular environment and cell surface, MT1-MMP may exert its physiological functions through its intracellular proteolytical activity, although more *in vivo* evidence is needed. However, how can MT1-MMP as a transmembrane protein with an extracellular catalytic domain cleave intracellular substrates? Eguchi et al. (2008) reported that the proline at the 5th position in the signal peptide of MMP3 might act as a helix breaker, causing a fraction of MMP3 to fail to enter the secretory pathway and remain in the cytosol. MT1-MMP also contains a proline residue at the 5th position in its signal peptide. Is it possible that the signal peptide of MT1-MMP is also inefficient, leading to the cytosol localization of the extracellular domain of some MT1-MMP? Alternatively, MT1-MMP may indirectly cleave intracellular substrate by activating some unknown proteinase. Further studies are required to elucidate these possibilities.

Recently, Chan et al. (2021) reported that hypoxia induced the interaction between MT1-MMP and hypoxia-inducible factor 2-α (HIF-2α) in nuclei of osteosarcoma U2OS and prostate carcinoma PC3 cells, but not in mesenchymal stem cells. They also reported cytoplasmic and nuclei localization of MT1-MMP in a patient sarcoma specimen (Chan et al., 2021). It will be of interest to see how MT1-MMP is transported to the nuclei and what is the physiological and pathophysiological significance of this intracellular location.

MT1-MMP may also act as a signaling protein in a proteolytic activity-independent manner (Gifford and Itoh, 2019). It has been reported that tissue inhibitor of metalloproteinases-2 (TIMP-2) bound to MT1-MMP and activated the ERK1/2 and AKT pathway, thereby regulating tumor apoptosis and growth (D'Alessio et al., 2008). Furthermore, Attur et al. (2020) reported that, after binding to TIMP-2, mutant MT1-MMP lacking the C-terminal cytoplasmic tail lost the activating ability, while the catalytic dead mutant MT1-MMP (E240A) still could activate ERK1/2, suggesting a non-proteolytic mechanism.

### MT1-MMP, lipoprotein metabolism, and atherosclerosis

Elevated levels of plasma cholesterol, especially low-density lipoprotein (LDL) cholesterol, are positively correlated to an increased risk of cardiovascular disease. Lowering circulating LDL cholesterol significantly reduces cardiovascular events. Approximately 70% of LDL cholesterol is removed by LDL receptor (LDLR) in the liver through receptor-mediated endocytosis. Mutations in LDLR cause familial hypercholesterolemia and increase the risk for atherosclerotic cardiovascular disease. The expression of LDLR is regulated by sterol regulatory element-binding protein 2 (SREBP-2) at the transcriptional level and by proprotein convertase subtilisin/kexin 9 (PCSK9) at the posttranslational level (Goldstein and Brown, 2015; Guo et al., 2020). In addition, the ectodomain of LDLR can be cleaved by proteinases with the release of the extracellular domain, namely soluble LDLR, to cell culture media and human plasma (Fischer et al., 1993; Begg et al., 2004; Shimohiro et al., 2014; Alabi et al., 2021).

Ectodomain shedding of LDLR was first reported in 1993 (Fischer et al., 1993). Since then, several lines of evidence have shown that serum levels of soluble LDLR are positively correlated with plasma LDL cholesterol levels (Begg et al., 2004; Shimohiro et al., 2014; Alabi et al., 2021). However, the proteinase responsible for LDLR shedding was unknown. Recently, we confirmed that plasma soluble LDLR levels are positively correlated with plasma levels of total and LDL cholesterol in the adult Chinese Han population (Alabi et al., 2021). Furthermore, we demonstrated that MT1-MMP is the proteinase mainly responsible for the ectodomain shedding of LDLR. Our data showed that knockdown of MT1-MMP expression in cultured human hepatocytes significantly reduced the levels of soluble LDLR in culture medium but increased cell surface LDLR abundance and enhanced LDL uptake. Consistently, knockdown of MT1-MMP in mouse liver significantly increased hepatic LDLR levels and concomitantly reduced plasma soluble LDLR levels, leading to a reduction in plasma levels of LDL cholesterol. Overexpression of MT1-MMP increased whereas knockdown of MT1-MMP expression ameliorated the development of atherosclerosis in a well-established mouse model of atherosclerosis, apolipoprotein E knockout (apoE^−/−^) mice (Alabi et al., 2021). These findings demonstrate the critical role of MT1-MMP in LDL cholesterol metabolism through promoting LDLR shedding.

In addition to pericellular substrates, MT1-MMP has been reported to cleave soluble plasma proteins, such as apoA-I and apoE (Hwang et al., 2004). ApoA-I is the major apolipoprotein and key structural element of plasma high-density lipoprotein (HDL). HDL enables cholesterol efflux from cholesterol-overloaded peripheral cells for the eventual excretion through reverse cholesterol transport, consequently inhibiting cholesterol accumulation in peripheral tissues and the formation of foam cells (Cukier et al., 2017; Yu et al., 2018). ApoE is primarily produced by the liver and macrophages and is a vital apolipoprotein for the catabolism of triglyceride-rich lipoproteins, such as VLDL and chylomicron remnants. It regulates cholesterol metabolism through interacting with LDLR and low-density lipoprotein receptor-related protein 1 (LRP1), thereby mediating clearance of triglyceride-rich lipoproteins (Actis Dato and Chiabrando, 2018; Ji et al., 2019). *ApoE^−/−^* mice display poor lipoprotein clearance and elevated levels of plasma cholesteryl ester-enriched lipoprotein particles, thereby promoting the development of atherosclerosis (Zhang et al., 1992; Veniant et al., 2001).

Park et al. (2008, 2011) employed *in vitro* cleavage assays combined with proteomic techniques and found that MT1-MMP cleaved apoA-I and apoE into smaller fragments, thus abrogating their functions. Considering the critical role of apoA-I and apoE in lipid metabolism, MT1-MMP may promote the development of hyperlipidemia by degrading apoA-I and apoE. Altogether, these findings suggest that MT1-MMP may reduce HDL levels through cleaving plasma apoA-I and increase LDL and remnant cholesterol levels via cleaving hepatic LDLR and plasma apoE, respectively, thereby increasing the risk of atherosclerosis. However, MT1-MMP-mediated cleavage of apoA-I and apoE needs to be verified *in vivo*.

### Macrophage MT1-MMP and atherosclerosis

Macrophage MT1-MMP has been shown to deteriorate the development of atherosclerosis, even though it does not directly affect plasma lipid levels like hepatic MT1-MMP. It is well documented that the rupture of atherosclerotic plaques causes 75% of cardiovascular events. Collagen confers tensile strength on the fibrous cap of the plaques, contributing to plaque stability and playing a crucial structural role in determining the vulnerability of advanced atherosclerotic plaques (Aikawa and Libby, 2004). As a membrane-tethered collagenase, MT1-MMP is localized in human atherosclerotic plaques (Rajavashisth et al., 1999b), and its expression is increased by nearly 26-fold in macrophage-derived foam cells within rabbit atherosclerotic plaques (Johnson et al., 2008). The expression of MT1-MMP is also high in foam cells in rupture-prone human carotid atherosclerotic plaques (Johnson et al., 2014). Given its key role in collagen hydrolysis and plaque localization, MT1-MMP is indispensably involved in collagen digestion and consequently promotes the instability of atherosclerotic plaques.

Inhibition of MT1-MMP by a neutralizing antibody reduces the collagen hydrolytic activity of macrophages (Johnson et al., 2008). Consistently, Schneider et al. (2008) found that bone marrow-derived macrophages from *Mmp14*^−/−^ mice displayed reduced collagenase activity compared with macrophages from the wild-type mice. Atherosclerotic plaques at the aortic arch of *Ldlr^−/−^* mice transplanted with *Mmp14^−/−^* bone marrow contained more collagen content than that of *Ldlr^−/−^* mice engrafted with *Mmp14^+/+^* bone marrow, even though the size of atherosclerotic plaques was comparable between the two groups (Schneider et al., 2008). These results suggest that macrophage MT1-MMP degrades collagen in plaques and therefore decreases the stability of plaques.

Arterial inflammation is another hallmark of atherosclerosis. Macrophage MT1-MMP plays an important role in the inflammatory response. Circulating monocytes migrate to the arterial intima, where they differentiate into macrophages to clear accumulated lipids, such as LDL and remnant cholesterol. Once becoming overloaded with lipids, macrophages are transformed into foam cells, initiating atherosclerosis. MT1-MMP is widely involved in inflammatory response and activation. The mRNA and protein levels of MT1-MMP are respectively increased by 24- and 30-fold in primary human monocytes during lung infection (Sathyamoorthy et al., 2015), suggesting the involvement of MT1-MMP in the inflammatory response in humans. MT1-MMP and MMP2 are highly expressed in bone marrow-derived human mesenchymal stem cells (hMSCs), the precursor of circulating monocytes. The ability of hMSCs to traverse reconstituted basement membranes is dramatically inhibited in the presence of TIMP-2 that can inhibit both MMP2 and MT1-MMP. Moreover, silencing TIMP-2 or MT1-MMP significantly enhances cell migration (Ries et al., 2007). These results suggest that the TIMP-2/MT1-MMP/MMP2 cascade may regulate the infiltration and migration of inflammatory precursor cells.

In the initial step of arterial response to injuries and angiogenesis, monocytes approach and attach to the inner layer of vasa vasorum at the vascular injury site. In the full-thickness skin wounds of mice lacking macrophage/monocyte MT1-MMP, the number of monocytes and macrophages was significantly reduced. Consistently, P-selectin, a pro-inflammatory cytokine that functions as a strong inflammatory cell adhesion molecule on the surfaces of activated endothelial cells, was reduced in ear lysate isolated from macrophage/monocyte MT1-MMP knockout mice. These findings suggest that MT1-MMP may activate P-selectin and thus enhance inflammatory monocyte adhesion (Klose et al., 2013). However, the exact underlying molecular mechanism remains elusive. Monocytes are differentiated into macrophages after traversing through the vascular endothelial basement membrane and can then undergo activation or polarization in the sub-endothelial space. M1 polarization of macrophages is one of the dominant causes of atherosclerosis and confers macrophages a high matrix-degradating ability. Inhibition of MT1-MMP by its monoclonal antibody DX-2400 stimulates polarization of bone marrow-derived macrophages to M2 phenotype (Kaneko et al., 2016), suggesting that MT1-MMP may promote macrophage M1 polarization. Moreover, many inflammatory cytokines, such as IL-1β and TNF-α, upregulate the expression of MT1-MMP in hMSCs, thereby strongly stimulating chemotactic migration through ECM (Ries et al., 2007). In summary, these findings suggest that MT1-MMP may function as a proinflammatory regulator to deteriorate the development of atherosclerosis.

### Vascular smooth muscle cell MT1-MMP and atherosclerosis

In contrast to hepatic and macrophage MT1-MMP, vascular smooth muscle cell (VSMC) MT1-MMP appears to protect against the development of atherosclerosis (Barnes et al., 2017). Knockout of VSMC MT1-MMP in *apoE^−/−^* (*Mmp14^−/−^*/*apoE^−/−^*) mice increased the size of atherosclerotic plaques and promoted the formation of aneurysm likely through promoting proinflammatory responses and enhancing smooth muscle proliferation. The lack of VSMC MT1-MMP had no significant effect on plasma cholesterol levels but markedly increased the proliferative potential of smooth muscle cells under a 3D spheroid but not a 2D culture condition. Thus, *Mmp14^−/−^*/*apoE^−/−^* mice developed a dysplastic structure in the arterial wall. Additionally, whole-genome transcriptome analyses showed that multiple proinflammatory genes were upregulated in VSMCs isolated from *apoE^−/−^* mice with the loss of VSMC MT1-MMP. Therefore, MT1-MMP silencing causes VSMC dysfunction, enhances their proliferation, and promotes a proinflammatory phenotype in VSMCs, leading to the development of proliferative atherosclerotic lesions.

In summary, these findings indicate the cell type-specific role of MT1-MMP in the development of atherosclerosis. Hepatic MT1-MMP promotes LDLR shedding, increasing plasma cholesterol levels and the development of atherosclerosis. On the other hand, macrophage MT1-MMP stimulates the fibrous cap’s turnover, thus reducing the stability of atherosclerotic plaques. Macrophage MT1-MMP may also modulate the inflammatory response probably through activating P-selectin, thereby promoting inflammatory cell adhesion. Conversely, VSMC MT1-MMP is important for maintaining normal VSMC function, likely through maintaining ECM homeostasis in VSMCs, thereby preventing proinflammatory actions in VSMC and reducing the development of atherosclerosis.

### MT1-MMP, adipocytes, and obesity

Dysregulation of lipid metabolism leads to many human diseases, such as diabetes, non-alcoholic fatty liver disease, and cardiovascular disease. Multiple risk factors are related to the initiation and development of dyslipidemia. Emerging evidence shows that ECM plays a critical role in establishing and maintaining tissue architecture and regulating many physiological and pathophysiological processes. Romani et al. (2019) reported that cellular lipid metabolism is sensitive to changes in ECM stiffness. A soft ECM activates the transcriptional activity of SREBP-1 and SREBP-2 through the Lipin-1/ARF1 pathway and then increases cholesterol biosynthesis and *de novo* lipogenesis, which leads to lipid accumulation in cells. MT1-MMP is essential for maintaining ECM homeostasis through proteolytic cleavage of ECM components, suggesting an important regulatory role of MT1-MMP in lipid metabolism.

Obesity is driven by excessive fat accumulation in adipocytes, leading to adipose tissue fibrosis, inflammation, and subsequent insulin resistance. Adipocytes and preadipocytes are surrounded by a dense fibril network composed of collagen, fibronectin, and laminin. ECM maintains adipose tissue architecture, forms a stable scaffold for adipocytes, and limits the hypertrophic growth of the cells (Alkhouli et al., 2013; Martinez-Santibanez et al., 2015). Dysfunctional adipose tissue expansion has been implicated in the development of metabolically unhealthy obesity (Fenech et al., 2015). MT1-MMP can digest interstitial type I, type II, and type III collagens but preferentially hydrolyzes type I collagen, the most abundant ECM component of adipocytes (Ohuchi et al., 1997; Van Doren et al., 2017). Together, these suggest that MT1-MMP may promote adipocytes’ hypertrophic expansion by cleaving the pericellular and perivascular matrix in adipose tissues.

In differentiating 3T3-L1 cells, MT1-MMP-dependent collagenolysis is critical for releasing the adipogenic histone mark, histone H3 acetylation at Lys9 (Sato-Kusubata et al., 2011). MT1-MMP is expressed in adipocytes, and its expression is increased in adipose tissues of diet-induced and genetic mouse models of obesity (Maquoi et al., 2002; Chavey et al., 2003). Several lines of evidence indicate that MT1-MMP can coordinate the differentiation of adipocytes (Chun et al., 2006; Chun et al., 2010; Li et al., 2020). Chun et al. (2006) reported that the loss of MT1-MMP did not affect the differentiation of preadipocytes into mature adipocytes under a 2D culture condition. Conversely, under a 3D culture condition or *in vivo*, the maturation of progenitors into adipocytes and healthy expansion of adipocytes required MT1-MMP-mediated turnover of the dense network of fibrillar collagen. Deficiency of MT1-MMP caused mini-adipocytes and lipodystrophy in mice (Chun et al., 2006). In addition to adipocytes, Lin et al. (2020) reported that MT1-MM-promoted adipogenesis of fibro/adipogenic progenitors and fatty infiltration in degenerative muscles, thereby deteriorating muscle repair. However, it is unclear whether this also depends on the MT1-MMP-mediated remodeling of the ECM.

MT1-MMP is also required for high-fat diet-induced rapid remodeling of the type I collagen fibril network in mouse adipose tissues (Chun et al., 2010). Heterozygous MT1-MMP knockout mice showed a significant reduction in adipogenic collagenolytic activity and protection against the diet-induced increase in fat mass. This implies the involvement of MT1-MMP in adipogenesis (Chun et al., 2010). Consistently, a variant in MT1-MMP (rs2236302) is positively correlated with obesity in women (Chun et al., 2010). It has been reported that adipogenesis and adipocyte inflammation increased the expression of TIMP-3, an endogenous inhibitor of MT1-MMP (Martinez-Santibanez et al., 2015). Synthetic MMP inhibitors could suppress lipid accumulation in human mesenchymal stem cells and adipogenesis (Bosco et al., 2017), suggesting a possible protective function of MT1-MMP in the development of obesity.

Recently, Li et al. (2020) reported that MT1-MMP in adipocytes affected the development of obesity in a stage-dependent manner. In an established obese model, expression of MT1-MMP enhanced degradation of collagen 6α3, leading to the production of endotrophin, which stimulated fibrosis, macrophage infiltration, and inflammation. As a result, MT1-MMP-overexpressing mice exhibited dysregulated lipid metabolism, insulin resistance, and reduced energy expenditure. However, in an early-stage obese model, overexpression of MT1-MMP in the adipose tissue released the mechanical stress of adipocytes through proteolytic cleavage of accumulated ECM proteins in the fat pad, resulting in healthy expansion of adipocytes and beneficial metabolic effects, such as reduced fibrosis and inflammation and improved lipid and glucose metabolism (Li et al., 2020). Collectively, these findings suggest that MT1-MMP plays a complex role in adipogenesis and obesity. MT1-MMP-dependent remodeling can modulate the shape and tension of adipocytes and adipocyte precursor cells in collagen-rich microenvironments and consequently affect adipose tissue expansion, which may delay the progression of early obesity but exacerbate advanced obesity.

## Regulation of MT1-MMP

### Regulation of MT1-MMP expression

MT1-MMP is expressed in most cells in various tissues and can cleave a wide range of substrates. Therefore, its activity is strictly regulated by multiple mechanisms at different levels. Epigenetically, transcription of MT1-MMP is suppressed by hypermethylation of the CpG islands in the proximal promoter region, trimethylation of histone H3K27, and acetylation of histone H3 (Chernov et al., 2009). At the transcriptional level, the promotor of the human *MMP14* gene does not contain the classic TATA box or binding sites of transcription factors (such as AP-1, AP-2, and TGF-α) commonly present in the promotor of other MMPs. Instead, it contains a non-canonical SP1 binding site and several putative binding sites of various transcriptional factors. SP1 is essential for the basal transcription of *MMP14.* Removal of the SP1-binding site markedly reduces the transcriptional activity of the promoter (Lohi et al., 2000). Many factors, including inflammation, hypoxia, oxidized LDL (oxLDL), growth factors, and cytokines (such as GM-CSF, TGF-β1, IL-1β, TNF-α, and IL-6), can stimulate *MMP14* transcription (Rajavashisth et al., 1999a, b; Tomita et al., 2000; Ray et al., 2004). However, the underlying mechanism is still elusive.

**Figure 2:**
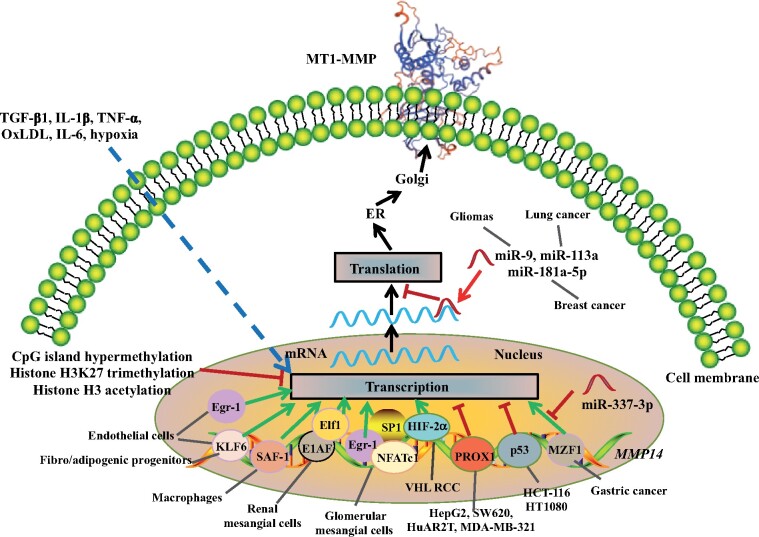
Regulation of MT1-MMP expression. TGF-β1, IL-1β, TNF-α, oxLDL, IL-6, and hypoxia increase whereas CpG island hypermethylation, histone H3K27 trimethylation, and histone H3 acetylation suppress the expression of MT1-MMP at the transcriptional level. Various transcriptional factors, such as Egr-1, KLF-6, SAF-1, Elf-1, MZF1, and HIF-2α, upregulate whereas PROX1 and p53 inhibit MT1-MMP transcription in a cell-type-dependent manner. MicroRNAs, such as miR-9, miR-113, and miR-181a-5p, target MT1-MMP mRNA and suppress its translation.

It is believed that specific transcriptional factors mediate *MMP14* transcription in a cell-type-specific manner (Figure 2). For example, the upregulated transcription of *MMP14* in endothelial cells during angiogenesis is mediated by early growth response 1 (Egr1) (Haas et al., 1999). Meanwhile, Gallardo-Vara et al. (2016) reported that Krüppel-like factor 6 (KLF6), a transcriptional factor that regulates the transcription of genes encoding proteins important for vascular remodeling and angiogenesis, could also bind to the promoter region of *MMP14* and upregulated its expression in endothelial cells. Haplodeficiency of *Klf6* in mice reduced MT1-MMP expression in the vasculature (Gallardo-Vara et al., 2016). In addition, KLF6 can activate the expression of MT1-MMP in fibro/adipogenic progenitors, promoting the differentiation of fibro/adipogenic progenitors to myofibroblasts or adipocytes (Lin et al., 2020). In macrophages, serum amyloid A activating factor-1 (SAF-1) transcriptionally upregulates MT1-MMP expression in atherosclerotic plaques, thus stimulating ECM degradation and plaque vulnerability (Ray et al., 2004). In renal mesangial cells, Elf1-1 and E1AF bind to the regulatory region 1 in the promoter region of *MMP14*. Two single-nucleotide polymorphisms –378T/C and –364G/T that are proximal to the regulatory region 1 reduce the expression of *MMP14* and are significantly associated with reduced risk of focal-segmental glomerulosclerosis (Munkert et al., 2009). Conversely, nuclear factor of activated T cells (NFAT) c1 instead of Elf1-1 or E1AF works together with SP1 and Egr1 to upregulate the transcription of *MMP14* in glomerular mesangial cells (Alfonso-Jaume et al., 2004). On the other hand, transcription factors HIF-2α and SP1 are responsible for the upregulation of *MMP14* expression in Von Hippel‒Lindau renal cell carcinoma (VHL RCC) (Petrella et al., 2005). In gastric cancer, myeloid zinc finger 1 (MZF1) upregulates *MMP14* expression, which can be competitively inhibited by miR-337-3p because this miRNA can bind to the same binding site of MZF1 on the promoter of *MMP14* (Zheng et al., 2016).

p53 can directly bind to but repress the activity of the *MMP14* promoter, thereby reducing *MMP14* expression at the transcriptional level in HCT-116 (a human colon cancer cell line) and HT1080 cells (human fibrosarcoma) (Cathcart et al., 2016; Gramolelli et al., 2018). Similarly, the transcriptional factor PROX1 can bind to the promoter region of *MMP14* and act as a transcriptional repressor to downregulate *MMP14* expression in the lymphatic system and various cell lines such as HepG2 (hepatoma), SW620 (colorectal carcinoma), HuAR2T (endothelial cells), and MDA-MB-321 (breast cancer) (Gramolelli et al., 2018). Additionally, miR-9, miR-113a, and miR-181a-5p can bind to the 3′-UTR of *MMP14* mRNA in the cytosol and reduce *MMP14* translation in gliomas, lung cancer, and breast cancer cells, respectively (Zhang et al., 2012; Xu and Wang, 2013; Li et al., 2015). miR-22-3p indirectly suppresses MT1-MMP expression via targeting the transcriptional factor KLF6 (Lin et al., 2020). In summary, these findings indicate the complexity and cell-type specificity of the regulation of *MMP14* expression at the transcription and posttranscriptional levels.

### Regulation of MT1-MMP activity

MT1-MMP is synthesized as an zymogen in the endoplasmic reticulum (ER). The latent MT1-MMP is activated through the cleavage of its pro-domain in the furin-recognition site between the pro-domain and the catalytic domain by the proprotein convertase activity of a furin-like enzyme in the *trans*-Golgi network (Rozanov and Strongin, 2003), even though a proprotein convertase-independent mechanism has been indicated (Rozanov et al., 2001). The active MT1-MMP is then delivered to the cell surface, where it enacts its physiological and pathophysiological functions. MT1-MMP can be efficiently inhibited by endogenous TIMP-2, TIMP-3, and TIMP-4 (Gifford and Itoh, 2019).

After fulfilling its functions, the cell surface MT1-MMP is inactivated via different mechanisms. The activity of MT1-MMP can be regulated through sequential autocatalytic cleavages on the peptide bond between Gly284 and Gly285 and between Ala255 and Ile256 to produce an 18-kDa soluble catalytic inert fragment and a 44-kDa inactive membrane-tethered remnant (Stanton et al., 1998; Lehti et al., 2000; Tam et al., 2002; Toth et al., 2002; Ludwig et al., 2008; Sato and Takino, 2010). The 44-kDa inactive form could act as a competitive inhibitor to suppress MT1-MMP-promoted collagenolysis and cell invasion when overexpressed in MDA-MB-231 cells (Tam et al., 2002). However, Cho et al. (2008) reported that overexpression of the 44-kDa inactive form in human fibrosarcoma cell line HT1080 reduced endocytosis of endogenous MT1-MMP, thereby promoting pro-MMP2 activation and cell migration. The reason for this discrepancy is unclear. It may be due to different cell types and/or experimental procedures used in the two studies. Nevertheless, the auto-cleavage process plays an important regulatory role in MT1-MMP-mediated proteolysis. Moreover, several lines of evidence showed that unknown proteinases could shed the ectodomain of MT1-MMP with a release of a ∼50-kDa soluble form into the extracellular milieu, such as culture medium, human sputum, bronchoalveolar lavage, and serum (Toth et al., 2002). The function of this soluble form is unclear. It will be of interest to see whether the 50-kDa soluble form can cleave MT1-MMP substrates in the circulation and/or at the final destination, because it contains the entire catalytic domain of MT1-MMP and potentially maintains its proteolytic activity.

### Tempo-spatial regulation of MT1-MMP activity

**Figure 3:**
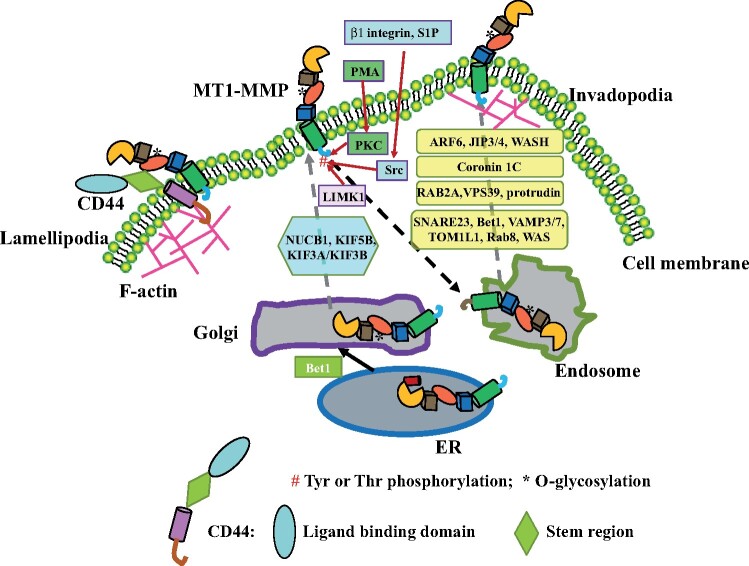
Trafficking of MT1-MMP. Pro-MT1-MMP is transported from the ER to the Golgi under the assistance of Bet1, where the prodomain is removed and O-glycosylation in HPX occurs. Mature MT1-MMP is then trafficked to the plasma membrane with the help of NUCB1, KIF5B, and/or KIA3A/KIF3B. The cell surface MT1-MMP can be redistributed to a specific microdomain in the plasma membrane through different mechanisms. CD44 can direct MT1-MMP to lamellipodia, and endocytosis and exocytosis can redirect MT1-MMP to invadopodia in cancer cells. Endocytosis of MT1-MMP can be stimulated by phosphorylation of Thr at the C-tail mediated by different kinases under different conditions. The transport of MT1-MMP from endosomes to invadopodia is regulated by various factors in different cancer cells.

The pericellular proteolytic cleavage activity of MT1-MMP is regulated by the level of cell surface-exposed MT1-MMP and the redistribution of the protein to specific microdomains in the plasma membrane (Figure 3). The *cis*-Golgi protein nucleobindin-1 (NUCB1) located in the *cis*-Golgi is crucial for the Ca^2+^-dependent transport of MT1-MMP along with the Golgi apparatus. Silencing of NUCB1 reduces the cell surface level of MT1-MMP (Pacheco-Fernandez et al., 2020). The microtubule-dependent motors KIF5B and KIF3A/KIF3B, on the other hand, mediate the transport of MT1-MMP along microtubules to the plasma membrane in primary human macrophages (Wiesner et al., 2010). On the cell surface, CD44 can direct MT1-MMP to lamellipodia by interacting with the hemopexin region of MT1-MMP via its stem region and associating with the F-actin cytoskeleton in lamellipodia via its cytoplasmic tail, promoting cell locomotion (Mori et al., 2002). Notably, MT1-MMP promotes CD44 shedding (Kajita et al., 2001). Mori et al. (2002) reported that the shedding process occurred after MT1-MMP was localized into lamellipodia. Interestingly, MT1-MMP appears not to promote CD44 shedding before reaching lamellipodia. Is it possible that the interaction of CD44 with F-actin in lamellipodia leads to a conformational change in CD44, which exposes the cleavage site of MT1-MMP on CD44? Additionally, given that the cleavage site of MT1-MMP on CD44 is within the stem region of CD44 that interacts with MT1-MMP (Kajita et al., 2001), is it possible that MT1-MMP-mediated CD44 shedding plays a role in removing MT1-MMP from the specific area after performing its function?

Cell surface MT1-MMP can be rapidly endocytosed through clathrin-mediated endocytosis in a dynamin-dependent manner or by caveolae-mediated endocytosis (Jiang et al., 2001; Remacle et al., 2003). This process is essential for redistributing MT1-MMP on the cell surface. Disruption of endocytosis of MT1-MMP does not impair MT1-MMP trafficking to the plasma membrane; instead, the cell surface levels of MT1-MMP are increased. However, targeting and enriching MT1-MMP to specific microdomains, such as invadopodia, are significantly reduced, leading to impaired cell migration (Williams and Coppolino, 2011). Endocytosis can also negatively regulate MT1-MMP activity via reducing cell surface MT1-MMP. For example, caveolin 1 interacts with MT1-MMP and promotes its endocytosis, suppressing the proteolytic activity of MT1-MMP (Labrecque et al., 2004; Kim and Chung, 2008).

After endocytosis, intracellular MT1-MMP mainly resides in the Rab7-positive late endosomes and can be redirected to invadopodia through exocytosis upon stimulation (Figure 3). In breast cancer cells, Wiskott‒Aldrich syndrome protein (WAS) and SCAR homolog recruit C-Jun NH2-terminal kinase-interacting protein 3 and 4 (JIP3/4) to MT1-MMP containing endosomes. JIP4 then interacts with the small GTPase ARF6, a plasma membrane protein, to facilitate endosome tubulogenesis for MT1-MMP exocytosis (Marchesin et al., 2015). Coronin 1C, a F-actin-binding protein required for the formation of invadopodia, can also mediate and direct the trafficking of endosomes containing MT1-MMP (Castagnino et al., 2018). RAB2A, on the other hand, regulates endosomal MT1-MMP trafficking to the plasma membrane in breast cancer cells via interacting with the endosome protein VPS39 and protrudin that mediates ER‒endosome contact (Kajiho et al., 2016; Pedersen et al., 2020). In addition, SNARE proteins SNARE23, Bet1, vesicle-association membrane 3 (VAMP3) and VAMP7, TOM1L1, and Rab8 GTPase are required to deliver MT1-MMP from late endosomes to invadopodia through exocytosis (Bravo-Cordero et al., 2007; Williams et al., 2014; Chevalier et al., 2016; Miyagawa et al., 2019).

Unlike in the noninvadopodial region of the plasma membrane, where surface MT1-MMP is rapidly cleared via endocytosis, MT1-MMP can more stably reside in invadopodia. Yu et al. (2012) reported that neural WAS facilitated the transport of MT1-MMP from late endosome to invadopodia and then docked it at invadopodia through tethering the C-terminal cytoplasmic tail of MT1-MMP to the invadopodial F-actin network. Conversely, WDFY2 inhibits v-SNARE VAMP3-facilitated exocytosis of MT1-MMP to invadopodia through docking VAMP3 on the phosphatidylinositol 3-phosphate-enriched domain in the endosomal membrane (Sneeggen et al., 2019).

Recently, Yu et al. (2021) reported that MT1-MMP mRNA also could be spatially regulated to translate MT1-MMP at a specific location. The authors found that agrin captured and stabilized ribonucleoprotein-containing granules and enriched MT1-MMP mRNA along the neurite, suggesting that MT1-MMP could be locally synthesized at the presynaptic terminals. Therefore, MT1-MMP activity can be regulated through local mRNA enrichment and protein synthesis even though the underlying mechanism for MT1-MMP mRNA intracellular trafficking is unknown. Overall, these findings indicate that endocytosis, vesicle transport, and fusion play an essential role in regulating the redistribution and function of MT1-MMP in specific microdomains of the plasma membrane. Targeting these pathways may introduce a novel avenue to inhibit MT1-MMP-mediated cell invasion and metastasis. However, due to the extensive physiological functions of endocytosis and membrane vesicle trafficking, more research is needed to determine whether and what proteins are specific to the redistribution of MT1-MMP in the plasma membrane.

### Posttranslational regulation of MT1-MMP

The function of MT1-MMP is also regulated by posttranslational modifications. MT1-MMP is predicted to be N-glycosylated on Asn at position 229 in the catalytic domain and position 311 in the first linker domain and O-glycosylated on Thr and Ser residues in the hinge region (Wu et al., 2004; Boon et al., 2016). Currently, there are no experimental data to support N-glycosylation in MT1-MMP. However, MT1-MMP is O-glycosylated on the hydroxyl group in the side chain of Thr and Ser residues in the hinge region. This modification is important for the recruitment of TIMP-2 and the subsequent activation of proMMP2 by MT1-MMP (Wu et al., 2004). O-glycosylation on different Thr and Ser residues of MT1-MMP can also block the self-cleavage site in the hinge region and then inhibit autocatalytic cleavage of MT1-MMP, thereby increasing its stability, cell surface levels, and proteolytic activity (Remacle et al., 2006; Kim et al., 2010).

MT1-MMP is phosphorylated at certain Tyr and Thr residues (Figure 3). Src kinase-dependent phosphorylation of Tyr573 is required for MT1-MMP-mediated migration of human umbilical vein endothelial cells and fibrosarcoma cells upon stimulation of sphingosine-1-phosphate (S1P) (Nyalendo et al., 2007). On the other hand, the LIM kinase-1 (LIMK1)-mediated phosphorylation of Tyr573 is necessary for MT1-MMP-promoted matrix degradation and cell invasion in breast carcinoma cells (Lagoutte et al., 2016). Phorbol 12-myristate 13-acetate (PMA) can activate protein kinase C, which phosphorylates Thr567 in MT1-MMP and leads to endocytosis of MT1-MMP in HeLa and human fibrosarcoma cells (Williams and Coppolino, 2011). In human breast cancer cell line MDA-MB-231 and fibrosarcoma cell line HT-1080, Thr567 is phosphorylated by Src kinase upon activation of β1 integrin, which enhances endocytosis of MT1-MMP and results in redistribution of MT1-MMP to invadopodia and subsequent cell invasion (Grafinger et al., 2020).

MT1-MMP is monoubiquitinated at Lys581 in the cytoplasmic tail. This posttranslational modification does not involve either lysosomal degradation or plasma membrane localization of the enzyme; instead, it regulates the trafficking of endocytosed MT1-MMP. Removal of the mono-ubiquitination site by replacing Lys581 with Ala causes accumulation of MT1-MMP in early endosomes and impairs MT1-MMP-mediated ECM degradation and cell invasion (Eisenach et al., 2012). Recently, Wang et al. (2020) reported that the TGF-β‒SMAD2/3 signaling pathway increased the expression of FBX06, a component of the ubiquitin E3 ligase, which subsequently promoted polyubiquitination and proteasomal degradation of MT1-MMP in cartilage. This consequently reduced MT1-MMP-dependent activation of MMP13 and then the development of osteoarthritis (Wang et al., 2020). It will be of interest to see whether FBX06 mediates monoubiquitinated at Lys581 of MT1-MMP.

MT1-MMP is also palmitoylated on Cys at position 574 in the cytoplasmic tail. This modification is not required for MT1-MMP-dependent activation of proMMP2; rather, it is necessary for the interaction of MT1-MMP with mu2 subunit of adaptor protein 2, regulating clathrin-mediated endocytosis of MT1-MMP and MT1-MMP-promoted cell migration (Anilkumar et al., 2005). In summary, the physiological and pathophysiological functions of MT1-MMP are regulated by various posttranslational modifications. Targeting these pathways may provide alternative ways to suppress MT1-MMP-promoted cell invasion specifically.

## Conclusion and Perspective

MT1-MMP is highly expressed in various cancer cells and promotes cancer metastasis and angiogenesis (Itoh, 2015; Amar et al., 2017). Emerging evidence shows that MT1-MMP can also regulate lipid metabolism through modifying the pericellular microenvironment and/or promoting protein shedding, thereby playing an essential physiological and pathophysiological role in the development of obesity and atherosclerosis. Collectively, these findings indicate that MT1-MMP acts as a common risk factor for cardiovascular disease and cancers, the two leading causes of global morbidity and mortality. Thus, inhibition of MT1-MMP is a very promising and valuable therapeutic target, because it can potentially reduce the risk of cancer metastasis, lower circulating LDL cholesterol levels, and increase atherosclerotic plaque stability. However, there are several challenges in developing MT1-MMP-based therapies.

### Tissue/cell type-specific role of MT1-MMP

MT1-MMP is widely expressed in various tissues with relatively high levels in the lung, kidneys, stomach, intestines, and skin (protein atlas). MT1-MMP is essential for postnatal development. Global *Mmp14^−/−^* knockout mice die at 3‒4 weeks after birth. However, mice with specific knockout of MT1-MMP in monocytes/macrophages, epidermis, or hepatocytes are indistinguishable from the wild-type littermates (Zigrino et al., 2012; Klose et al., 2013). Knockout of hepatic MT1-MMP also does not cause liver damage, indicating a negligible role of MT1-MMP in the development of the liver (Alabi et al., 2021), whereas VSMC MT1-MMP plays an important role in the development of the blood vessel wall (Barnes et al., 2017), indicating a tissue-specific function of the proteinase. Thus, a specific knockout of *Mmp14* in different tissues is required for elucidating its exact tissue-specific role.

### Tissue-specific targeting

The pathophysiological role of MT1-MMP appears to be tissue/cell-type dependent. The loss of hepatic or macrophage MT1-MMP alleviates the risk of atherosclerosis; however, globally haplodeficiency of MT1-MMP increases atherosclerotic lesion area in *apoE^−/−^* mice (Barnes et al., 2017). Thus, cell type/tissue-specific manipulation of MT1-MMP expression is required for therapeutic intervention to avoid unwanted side effects. For example, N-acetylgalactosamine (GalNAc) can bind to hepatic asialoglycoprotein receptor, leading to a rapid uptake of siRNA (Springer and Dowdy, 2018). This technology has been successfully used to silence hepatic PCSK9 expression in a clinical trial (Cupido and Kastelein, 2020). Thus, we can use GalNAc-conjugated siRNA to specifically inhibit hepatic MT1-MMP for lowering plasma LDL cholesterol levels, thereby reducing the risk of atherosclerosis for patients who are intolerant to or cannot be effectively managed by existing therapies.

### MT1-MMP specific inhibitor

The MMP family contains 23 members. Therefore, to avoid off-target effects, a highly specific and selective MT1-MMP inhibitor is required. The X-ray crystallographic structure of the full-length MT1-MMP or its intact extracellular domain is currently unavailable. The catalytic domain of MT1-MMP is highly conserved among MMPs (Fernandez-Catalan et al., 1998; Grossman et al., 2010; Decaneto et al., 2017). Therefore, it is challenging to develop inhibitors specifically inhibiting the proteolytic activity of MT1-MMP by targeting the catalytic domain. However, exosites of MMPs, which are outside the catalytic core domain and less conserved, are involved in substrate selection and binding. For example, the hemopexin domain of MT1-MMP consists of four blades of the β-propeller that commonly exist in the hemopexin domain of other MMPs and one MT1-MMP unique structure composed of two very short β-sheets connected by a bulged loop (Tochowicz et al., 2011). The hemopexin domain can directly bind to type I collagen (Tam et al., 2004). The blade IV is essential for homodimerization of MT1-MMP, which is essential for cleaving type I collagen fibers on the cell surface (Tochowicz et al., 2011; Udi et al., 2015). The blade I is necessary for the interaction of MT1-MMP with CD44, a process important for regulating cell migration (Zarrabi et al., 2011). Therefore, the hemopexin domain has been identified as a promising target to inhibit MT1-MMP-mediated cellular invasion specifically. Indeed, a peptide targeting this region has been shown to specifically inhibit the proteolytic activity of MT1-MMP and then cancer cell migration without a detectable inhibitory effect on other MMPs, such as MMP1, MMP3, MMP7‒MMP13, MMP20, MT4-MMP, and MT2-MMP. A small inhibitor, NSC405020 (3,4-dichloro-N-(1-methylbutyl) benzamide), which targets the HPX of MT1-MMP, also can inhibit tumor growth (Fields, 2015). Additionally, MT1-MMP contains a specific MT-loop (^163^PYAPIREG^170^) in the catalytic domain. The MT-loop is flexible and displays different conformations in MT1-MMP complexed with or without TIMPs or among different MT-MMPs (Fernandez-Catalan et al., 1998; Lang et al., 2004; Grossman et al., 2010; Decaneto et al., 2017), indicating a specific role of this loop in MT1-MMP function. Removal of the MT-loop does not significantly affect the proteolytic activity of MT1-MMP but reduces the association of MT1-MMP with TIMP-2 and pro-MMP2 activation (English et al., 2001). The MT-loop is also crucial for MT1-MMP-mediated cellular invasion probably through interacting with β1 integrin-containing cell adhesion complexes (Woskowicz et al., 2013). Thus, the specificity and accessibility of the MT-loop represent a potential exosite target for the development of selective MT1-MMP inhibitors. An antibody against the MT-loop of MT1-MMP can block pro-MMP2 binding to MT1-MMP and suppress pro-MMP2 activation (Shiryaev et al., 2013). Botkjaer et al. (2016) also developed antibody fragments that specifically target a region outside the active site cleft of MT1-MMP with a high affinity and could inhibit MT1-MMP-mediated metastasis *in vivo*. Therefore, drugs targeting exosites of MT1-MMP may worth exploring in future studies. However, this requires a thorough understanding of the structure‒function relationship of MT1-MMP.

### Regulation of MT1-MMP expression in tissues other than tumors

The expression and function of MT1-MMP are regulated by different mechanisms at multiple levels. Targeting these pathways may provide an alternative way to inhibit MT1-MMP function. However, our understanding of these mechanisms is mostly based on studies on cancer cells. The relevance of these mechanisms under physiological and other pathophysiological conditions is not well understood. Most recently, Alonso-Herranz et al. (2020) reported that the expression of macrophage MT1-MMP was increased after myocardial infarction, which activated TGF-β1 and then the SMAD2 signaling pathway in endothelial cells. This process promoted endothelial-to-mesenchymal transition and subsequent adverse cardiac remodeling after myocardial infarction. Silencing macrophage MT1-MMP could prevent this adverse effect (Alonso-Herranz et al., 2020). However, the mechanism underlying elevated expression of MT1-MMP in macrophages remains elusive. It has been reported that SAF-1 activated MT1-MMP transcription in macrophages in atherosclerotic plaques (Ray et al., 2004). It will be of interest to see whether SAF-1 regulates macrophage MT1-MMP expression in the heart after myocardial infarction. Nevertheless, more studies are needed to dissect the mechanisms regulating MT1-MMP expression and functions under physiological and pathophysiological conditions.
